# Multi-omics reveals glutinous rice varieties shape Baijiu flavor via microbial and metabolic modulation

**DOI:** 10.3389/fmicb.2025.1721127

**Published:** 2026-01-16

**Authors:** Jia Zeng, Lijuan Gong, Shuai Qin, Pengpeng Fang, Fu Shu, Wuhan Zhang, Yi Zhou, Xinpeng Li, Qiang He, Pingyong Sun, Huafeng Deng

**Affiliations:** 1 Longping Agricultural College, Hunan University, Changsha, China; 2 Sichuan Engineering Technology Research Center for Liquor-Making Grains, Sichuan University of Science and Engineering, Yibin, China; 3 Yuelushan Laboratory, Changsha, China; 4 State Key Laboratory of Hybrid Rice, Hunan Hybrid Rice Research Center, Changsha, China

**Keywords:** glutinous rice, brewing-specific rice, metagenomics, metaproteomics, flavor quality

## Abstract

**Introduction:**

Glutinous rice significantly influences Baijiu flavor, yet standardized brewing-specific indicators are lacking.

**Methods:**

In this study, metagenomic, metaproteomic, and non-targeted GC–MS analyses of *Zaopei*, along with HS-SPME-GC–MS analysis of Baijiu, were used to compare the effects of three glutinous rice varieties with distinct nutritional profiles on microbial diversity and flavor formation.

**Results:**

The *Wuliangye*-specific variety *Dajiugu*, with high sucrose, high amino acids, and low fatty acids, promoted early growth and metabolic activity of *Saccharomycopsis*, *Enterobacter*, and *Klebsiella*. Functional genera such as *Saccharopolyspora*, *Pediococcus*, and *Clostridium* enhanced fatty acid and amino acid accumulation in *Zaopei* and increased ethyl acetate, 4-vinylphenol, and dimethyl trisulfide in Baijiu.

**Discussion:**

These findings highlight the pivotal role of glutinous rice variety in shaping Baijiu flavor and offer a scientific basis for breeding brewing-specific glutinous rice.

## 1 Introduction

Glutinous rice, characterized by extremely low amylose content, is highly suitable for saccharification and is widely used as a key grain substrate in the production of Chinese Baijiu, Huangjiu, and glutinous rice sweet wine. Among these, Baijiu dominates the Chinese liquor market, comprising five major aroma types: strong, soy sauce, light, sweet honey, and miscellaneous, with strong-aroma Baijiu accounting for 51% of total market share and reaching a production volume of 7.156 billion liters in 2021 (Liu et al., 2023). Baijiu is produced from grains, *Jiuqu* (the primary microbial inoculum), and water. After steaming, the grains absorb water and are mixed with *Jiuqu* and rice husks to form *Zaopei* (fermented grain mixture), which undergoes fermentation and subsequent distillation. Microorganisms in *Zaopei* play a pivotal role in flavor formation. For instance, *Weissella* correlates positively with capric acid and isoamyl alcohol; *Rhizopus* with ethyl propionate, ethyl laurate, and ethyl butyrate; and *Pediococcus* with ethyl laurate and acetic acid (Chen et al., 2020).

Previous studies on flavor-microbe associations have largely focused on *Jiuqu* microbiota. However, microbial dominance during fermentation is strongly influenced by the nutrient composition of the grain substrate, particularly the availability of carbon and nitrogen sources. While *Jiuqu* supplies the initial microbial community, microbial composition shifts during fermentation based on raw material differences, leading to the emergence of different dominant taxa. Flavor differences have been reported among different grain types (Taylor et al., 2013), among rice varieties with high and low amylose contents (Park et al., 2023), and between glutinous and non-glutinous rice (Liu et al., 2023). Amylose content is a key factor determining rice gelatinization, retrogradation, and cooking quality, directly influencing swelling, digestibility, and viscosity (Zhou et al., 2025). However, glutinous rice contains virtually no amylose, resulting in minimal variation in starch physicochemical properties among varieties. Current breeding standards for brewing-specific glutinous rice focus on parameters such as starch content, gelatinization temperature, and retrogradation value. As starch serves as the main carbon source for microbes, lower gelatinization temperatures reduce steaming costs, while excessive retrogradation after cooling can form *β*-starch, which is poorly utilized by microorganisms. Therefore, it is necessary to explore alternative nutritional and physicochemical traits beyond starch to support the development of glutinous rice varieties optimized for Baijiu brewing.

In addition to starch, glutinous brown rice contains other nutritional components such as proteins, lipids, and phenolics. Although some studies have suggested that microbial succession driven by raw materials may not be strongly correlated with Baijiu flavor (Xu et al., 2018), the degradation products of starch, protein, and fat, such as glucose, amino acids, and fatty acids, can serve as microbial substrates and contribute to the formation of distinct aroma compounds (Yang et al., 2020). For example, L-phenylalanine levels in raw materials have been positively associated with 2-phenylethanol production (Pineda et al., 2012). To enhance flavor diversity in Huangjiu, researchers have explored alternative grains; Zhao et al. (2024) reported that blending protein-rich Chinese wild rice with glutinous rice, despite reducing ethanol content, significantly increases total phenolics and aroma intensities such as fruity, honey-like, caramel, herbal, and smoky notes. Beyond traditional grains, unconventional ingredients like loquat leaves, oats, highland barley, and buckwheat have also been used in Huangjiu fermentation (Yang et al., 2020). Xu et al. (2018) further found that sorghum varieties with different tannin levels resulted in distinct microbial-flavor associations: *Blautia*, *Collinsella*, and *Bifidobacterium* are enriched under high-tannin conditions and correlat with elevated ester content, while *Stenotrophomonas*, *Bdellovibrio*, and *Solibacillus* dominate in low-tannin fermentations.

Our previous research revealed that Chinese indigenous glutinous rice, having undergone minimal domestication, exhibits rich genetic diversity. Among numerous accessions, three representative varieties, namely Dajiugu (DJG), Dafengnuo (DFN), and TeAnuo (TAN), exhibit significant differences in nutritional composition and starch physicochemical properties. DJG, a long-used brewing-specific glutinous rice for strong-aroma Baijiu by Wuliangye, presents ideal fermentation characteristics, though its underlying mechanisms remain unclear. To better elucidate the relationships among glutinous rice composition, microbial dynamics in *Zaopei*, and Baijiu flavor formation, we performed integrated multi-omics analyses, including metagenomics, metaproteomics, liquid chromatography–mass spectrometer (LC–MS) based metabolomics, and headspace solid-phase microextraction coupled with gas chromatography–mass spectrometry (HS–SPME–GC–MS), on both *Zaopei* and final liquor samples. This study provides new insights into how glutinous rice varietal differences influence microbial ecology and flavor development during Baijiu fermentation.

## 2 Materials and methods

### 2.1 Seed physicochemical testing

All three glutinous rice varieties were Chinese-bred cultivars grown at the Hybrid Rice Research Center (Hunan, China). Among them, DJG is a brewing-specific glutinous rice variety, whereas DFN and TAN are glutinous rice varieties used for food and processing purposes. Glutinous rice seeds were harvested, air-dried, and dehulled. Starch properties and gelatinization characteristics were measured following NY/T 83–2017 standards by Shanghai Sanshu Co., Ltd. The brown rice was milled and sieved through an 80 mesh screen for further analysis.

Fatty acids, soluble sugars, and amino acids were quantified using calibration curves established with commercial standards (purchased from Shanghai ANPEL and YuanYe Bio). Fatty acid analysis followed the method described by Li et al. (2015). Samples were subjected to acid hydrolysis, extracted with petroleum ether and diethyl ether, followed by saponification and methyl esterification, and then analyzed by gas chromatography–flame ionization detector (GC–FID) using a DB-225 capillary column (Agilent Technologies, United States). Soluble sugars were determined according to the national standard GB/T 5009.8–2016. After extraction, centrifugation, and filtration, samples were analyzed by high performance liquid chromatography–refractive index detector (HPLC–RID) using an Athena NH2–RP (II) column. Amino acids were analyzed following the method of Liang et al. (2020). Samples were treated with formic acid/acetonitrile for protein precipitation, centrifuged, and reconstituted, and then analyzed by Ultra Performance Liquid Chromatography (UPLC)–MS/MS with a Premier HSS T3 column (Waters Corporation, United States) under gradient elution conditions.

Total phenolics in the seeds were determined using a Fluorescence Microplate Reader (Infinite F200, Tecan Group Ltd., Switzerland), based on their reduction of phosphomolybdic–phosphotungstic acid under alkaline conditions to form a blue complex with an absorbance maximum at 760 nm.

### 2.2 Xiaoqu Baijiu brewing procedure using glutinous rice

The brewing experiments were conducted at the Brewing Grain Engineering Research Center, Sichuan University of Science and Engineering. For each rice variety, three independent biological replicates (1.0 kg per replicate) were prepared. All replicates were processed under identical brewing conditions using the same batch of Sichuan Huaxi *Xiaoqu* and the same fermentation equipment. The grains were washed with boiling water under agitation, then soaked in water at 50 °C for 12 h. After draining, the samples were steamed for 90 min, cooled to room temperature with sterile water on a clean bench, and inoculated with 0.6% Sichuan Huaxi *Xiaoqu*. The inoculated grains were mixed thoroughly and transferred to sterile ceramic jars. A small portion was placed into sterile Erlenmeyer flasks.

All saccharification and fermentation processes were performed in a controlled-temperature incubator at 30 °C (±0.5 °C) with relative humidity maintained at 65–70%. After 24 h of saccharification in a sterile incubator, rice husks were added and mixed. Samples were sealed and fermented at 30 °C for 8 days (Figure 3F). *Zaopei* from the Erlenmeyer flasks was collected on days 0, 4, and 8 for physicochemical analysis, following the procedures specified in “The General Analysis Method of Solid-State Fermented Grains” (T/CBJ 004–2018).

After fermentation, *Zaopei* in ceramic jars was sampled from multiple points for microbial multi-omics analysis. The remaining *Zaopei* was distilled, and the Baijiu was collected after discarding the initial and final fractions.

### 2.3 DNA extraction and metagenomic analysis of *Zaopei*

Total microbial DNA was extracted from *Zaopei* samples collected on day 8 of fermentation, with three biological replicates for each sample, using the MGIEasy Microbiome DNA & RNA Extraction Kit (MGI Tech, China). DNA concentration was measured using a spectrophotometer (NanoDrop 2000, Thermo Fisher Scientific, United States), and DNA integrity was assessed by agarose gel electrophoresis.

Genomic DNA was fragmented to an average length of 200–400 bp using a focused ultrasonicator (Covaris M220, USA), followed by library construction. Paired-end sequencing (PE150) was performed on the BGISEQ-500 platform. Raw metagenomic data were deposited in the National Genomics Data Center (Accession No.: PRJCA045750). Host genome sequences (rice cultivar Nipponbare, IRGSP-1.0) were removed prior to downstream analysis by mapping reads to the reference genome using Bowtie2.

Differential abundance analysis between groups was conducted using a threshold of fold change > 2 and *p*-value < 0.05. Functional annotation was conducted by aligning non-redundant gene sets against the NR, KEGG, eggNOG, COG, and Swiss-Prot databases using DIAMOND (v0.9.24). Taxonomic classification was performed with Kraken2 (v2.1.2), and species abundance was estimated using Bracken (v2.6.0). Microbial diversity was assessed via alpha diversity indices (Chao1, Shannon, Simpson) and beta diversity metrics (Bray–Curtis distance, Jensen–Shannon divergence).

### 2.4 Protein extraction and metaproteomic analysis of *Zaopei*

Total proteins were extracted from day 8 *Zaopei* samples, with three biological replicates for each sample, using DNeasy PowerSoil Protein Kit (QIAGEN, Germany). Protein concentration was determined using the Bradford assay, and protein quality was assessed by SDS-PAGE. Proteins were digested with trypsin to obtain peptides.

For peptide fractionation, high-pH reversed-phase liquid chromatography was performed using an HPLC system (LC–20 AD, Shimadzu Corporation, Japan) equipped with a 5 μm, 4.6 × 250 mm C18 column (Gemini C18, Phenomenex Inc., United States). Further separation was conducted using a ultra-high performance liquid chromatography (UHPLC) system (UltiMate 3,000, Thermo Fisher Scientific, USA), coupled to an Orbitrap Tribrid mass spectrometer (Fusion Lumos, Thermo Fisher Scientific, USA) for data-dependent acquisition (DDA) and data-independent acquisition (DIA) analyses.

A reference protein database was constructed based on metagenomic data. Raw spectra were searched using X! Tandem^1^ with one to two iterations. The resulting database files were imported into MaxQuant^2^ for protein identification and quantification. Proteins with a fold change >2 and *p*-value <0.05 were considered significantly different. Differential protein analysis and enrichment were conducted using the MSstats package in R.

Protein annotation was performed by aligning sequences to NR (202004), eggNOG (v5.0), COG (2014–11), Swiss-Prot (2020–02), KEGG (v89.1), and STRING (v11.0) databases using DIAMOND. Taxonomic annotation was performed with MEGAN (v4.6).

### 2.5 Untargeted LC–MS metabolomic analysis of *Zaopei*

On day 8 of fermentation, *Zaopei* samples were extracted using a solvent containing internal standards (d3-leucine, ^13^C₉-phenylalanine, d5-tryptophan, and ^13^C₃-progesterone), with three biological replicates for each sample. After evaporation to dryness, the residues were reconstituted in methanol:water (1:1, v/v) and transferred to vials for analysis. Metabolite profiling was conducted using a Q Exactive HF mass spectrometer (Thermo Fisher Scientific, United States) to acquire full-scan MS and MS/MS data.

Raw data were processed using Compound Discoverer 3.3 (Thermo Fisher Scientific, United States), and metabolites were annotated through BMDB (BGI Metabolome Database), mzCloud, and the ChemSpider online database. For the key differential metabolites identified in the subsequent analyses, high-confidence annotation was achieved by matching the experimental MS/MS spectra against mzCloud reference libraries using accurate mass (±5 ppm), retention time window, and characteristic fragment ions. Spectra were manually inspected to confirm key diagnostic fragments. Peak area data and compound identifications were subjected to quality control, principal component analysis (PCA), and differential analysis using a threshold of fold change >2 and *p*-value <0.05.

Enrichment analysis of differential metabolites was performed using the KEGG database, with reference to the genomes of the top 10 dominant species identified from metagenomic data. The top five enriched pathways (lowest *p*-values) were visualized as pathway interaction networks.

### 2.6 Determination of volatile organic compounds in Baijiu

The distilled Baijiu samples were analyzed using HS–SPME–GC–MS on a GC–MS system (Agilent Technologies 7890B GC, 5977A MSD, United States). Compound identification and quantification were performed based on a proprietary database developed by FlavorDB Metabolomics. Only compounds with a coefficient of variation (CV) less than 0.5 were retained. 1,4-Di(methyl-d3)benzene-d4 was used as the internal standard. A working solution of the internal standard was prepared at a concentration of 1 μg/mL, and 10 μL of this solution was added to each sample prior to extraction. The concentration of each volatile compound was calculated using the following equation:


$$X_{i}=\frac{V_{s}\times C_{s}}{V}\times \frac{I_{i}}{I_{s}}\times 10^{-3}$$

where *X_i_* is the concentration of compound *i* in the sample (μg/mL); *V_s_* is the volume of internal standard added (μL); *C_s_* is the concentration of the internal standard (μg/mL); *V* is the volume of the sample (mL); *I_s_* is the peak area of the internal standard; and *I_i_* is the peak area of compound *i*. Relative concentrations of VOCs were estimated by semi-quantitative analysis using internal standard calibration. The relative odor activity value (rOAV) for each compound was calculated as:


$${\text{rOAV}}_{i}=\frac{C_{i}}{T_{i}}$$

where rOAV*_i_* is the relative odor activity value of compound *i*, *C_i_* is its relative concentration (μg/mL), which was obtained from values reported in the literature, and *T_i_* is its odor threshold (μg/mL). Compounds with rOAV ≥ 1 were considered key aroma contributors. Compound classification was primarily based on information from the following databases: PubChem, Chem960, and ClassyFire. Aromatic descriptions of the compounds were obtained from The Good Scents Company, Perflavory, Odour, and Food Flavor Lab. Differential VOCs were screened using the criteria: fold change ≥ 2, *p*-value < 0.05, and VIP > 1. Principal component analysis (PCA) and Venn diagrams were applied to compare sample groups. Quantitative data were scaled using unit variance scaling for hierarchical clustering analysis. Based on sensory annotations of differential VOCs, the top 10 sensory attributes (by number of annotated compounds) were visualized using radar plots and Sankey diagrams. For each sensory attribute, the top five VOCs with the highest fold changes were included in the Sankey diagram.

### 2.7 Statistical analysis and visualization

Significant differences in physicochemical parameters among the three *Zaopei* samples were assessed using one-way ANOVA followed by Tukey’s *post hoc* test (*p* < 0.05). For metagenomic and metaproteomic diversity analyses, alpha diversity indices (Chao1, Shannon, Simpson) were compared using the Kruskal–Wallis test, and beta diversity was evaluated using PERMANOVA (Adonis) based on Bray–Curtis and Jensen–Shannon distance matrices. All statistical analyses were performed using SPSS. All chromatographic data were processed using calibration curves, and compound concentrations were calculated based on peak areas. Metabolite expression data were log₂-transformed and *z*-score normalized for hierarchical clustering based on Euclidean distance. Network diagrams were generated using Cytoscape. Bar charts, line graphs, and basic statistical analyses were performed using GraphPad Prism 8. All other visualizations were generated using R packages, and final figure assembly was completed in Adobe Illustrator.

## 3 Results and discussion

### 3.1 Physicochemical differences among glutinous rice varieties

**Figure 1:**
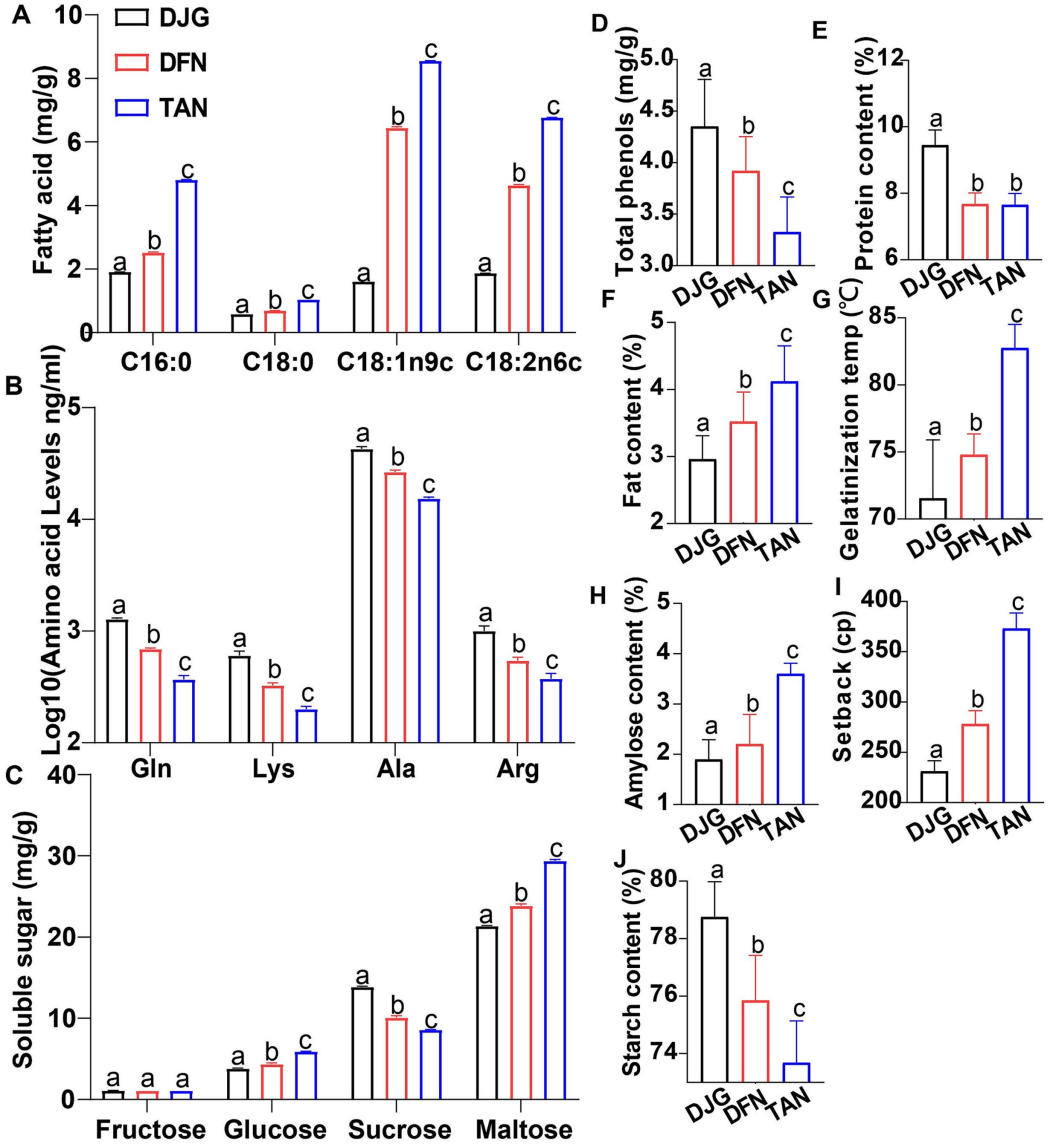
Physicochemical properties of brown rice from different glutinous rice varieties: **(A)** Four major fatty acids; **(B)** Four significantly different amino acids; **(C)** Four major soluble sugars; **(D)** Total phenolic content; **(E)** Protein content; **(F)** Fat content; **(G)** Gelatinization temperature; **(H)** Amylose content; **(I)** RVA setback value; **(J)** Starch content. All data were obtained from two consecutive years of cultivation. For each variety, three individual plants were analyzed. Error bars represent ±SD (*n* = 3). Significant differences were determined by letter-based pairwise comparison.

The three glutinous rice varieties exhibited stepwise differences in starch gelatinization properties, protein content, and fat content (Figures 1E–J). DJG has a lower gelatinization temperature, lower amylose content, and lower retrogradation value, as well as a higher starch content, all of which are favorable traits for steaming and brewing (Figures 1G–J). To further characterize compositional differences among brown rice samples, levels of free fatty acids, amino acids, soluble sugars, and total phenolics were analyzed. Several major C16 and C18 fatty acids, including linoleic acid, were proportional to total fat content (Figure 1A), displaying a gradient distribution across DJG, DFN, and TAN. Other fatty acids were either present at low levels or undetected. While oxidized fatty acids contribute to aroma compounds such as aldehydes, ketones, and acids, free long-chain fatty acids such as palmitic, stearic, and linoleic acids may inhibit microbial activity by disrupting cell membrane integrity (Obukhova and Murzina, 2024). Additionally, these fatty acids may form stable complexes with amylose, significantly reducing starch gelatinization and saccharification efficiency (Arik Kibar et al., 2014).

The initial amino acid concentrations markedly influence microbial species composition and growth rate. Strong positive correlations have been reported between amino acid levels and the formation of fusel alcohols and medium-chain fatty acid ethyl esters (Kang et al., 2014). Four amino acids showed significant differences among the varieties (Figure 1B). Lysine and arginine serve as key nitrogen sources for yeast growth, while glutamine promotes the production of ethyl isovalerate, ethyl phenylacetate, ethyl nonanoate, methyl decanoate, diethyl succinate, phenylethanol, and isobutanol during fermentation (Zhu et al., 2023). However, branched-chain amino acids (BCAAs), including leucine, isoleucine, and valine, which are preferentially utilized by microbes, showed no significant differences among varieties. This suggests that early-stage microbial metabolism in different rice varieties is not primarily driven by the Ehrlich pathway for nitrogen catabolism.

**Figure 2:**
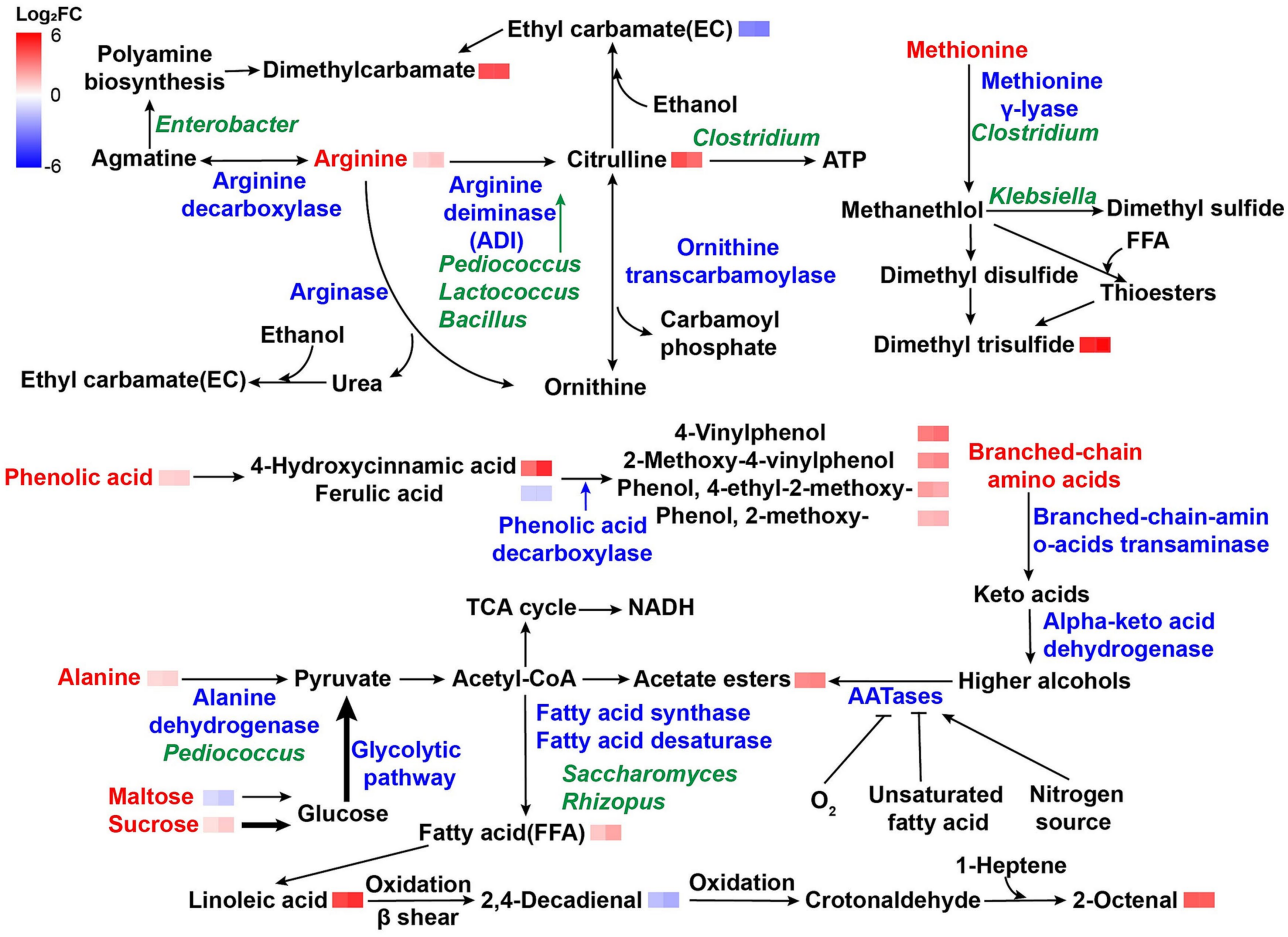
Metabolic pathways of grain-derived compounds in dominant microbial taxa. The heatmap blocks to the right of each metabolite indicate its fold change (log-transformed) in DJG compared with DFN (left) and TAN (right). Red labels represent compounds originating from raw materials, blue labels indicate enzymes, and green labels denote microbial genera involved in the corresponding reactions.

Soluble sugar content showed a different pattern. While sucrose levels were highest in DJG, other major sugars exhibited the opposite trend. Brown rice samples primarily contained sucrose and maltose (Figure 1C). Glucose, fructose, sucrose, and maltose are the dominant sugars in rice. In addition to these, Hu et al. (2023) also detected raffinose, with maltose being the least abundant and sucrose the most. Compared to maltose, sucrose is less likely to generate bitterness in sweet fermented rice products (Jiang et al., 2002). Some microbes, such as lactic acid bacteria, can directly uptake and hydrolyze sucrose intracellularly, whereas maltose requires specific transport proteins (e.g., MALx1, MalR) for cellular import and metabolism, making it less accessible (Andersson and Rådström, 2002). Therefore, in high-sucrose varieties like DJG, microbes can readily obtain fermentable substrates through rapid sucrose hydrolysis, even when maltose and glucose levels were relatively low (Figure 2).

### 3.2 Changes in physicochemical parameters of *Zaopei* during fermentation

**Figure 3:**
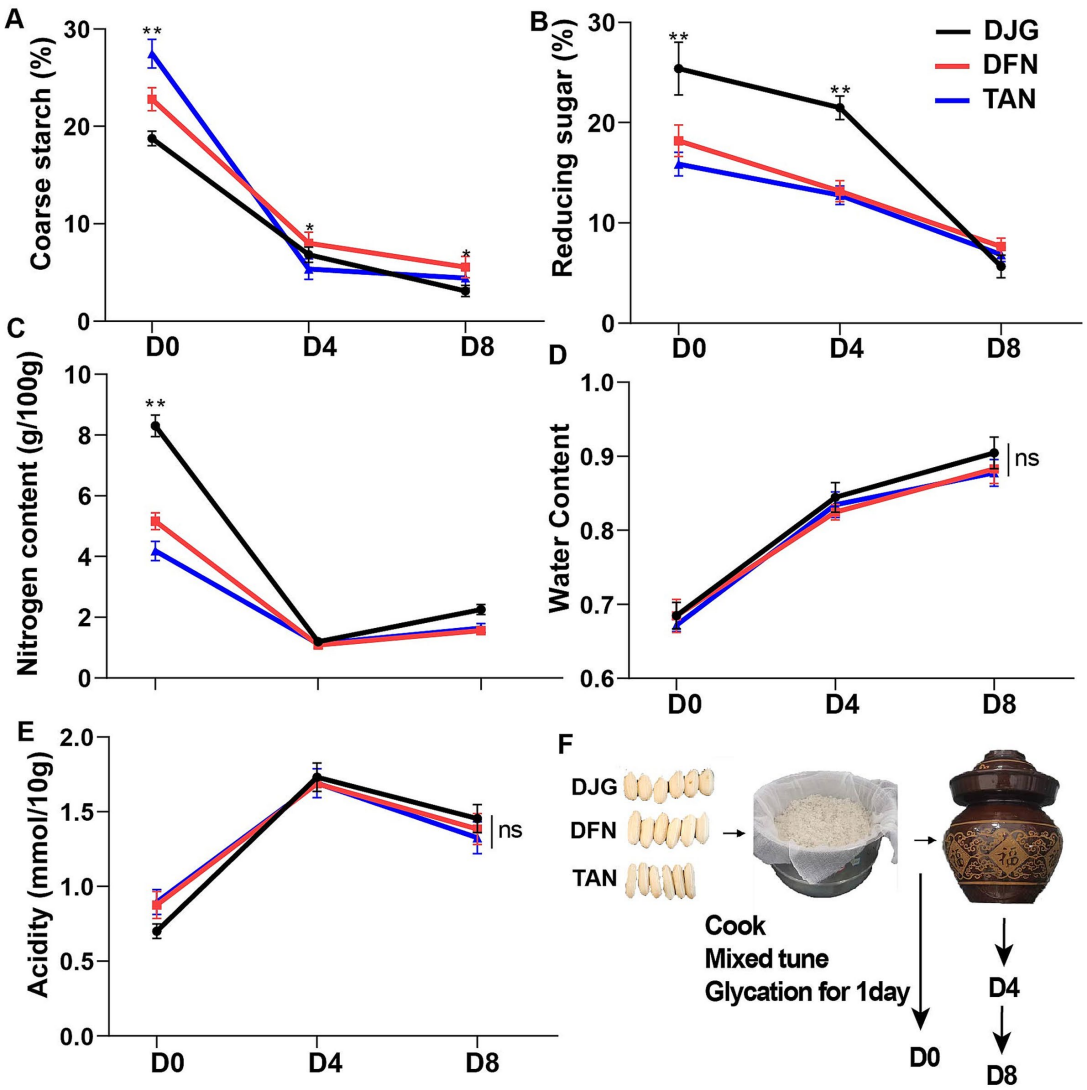
Physicochemical parameters of *Zaopei* during saccharification and fermentation. **(A)** Crude starch content; **(B)** reducing sugar content; **(C)** nitrogen content; **(D)** moisture content; **(E)** acidity; **(F)** schematic of the fermentation process. Error bars represent ±SD (*n* = 3). Statistical significance: ^*^p < 0.05; ^**^p < 0.01.

The error bars indicated low dispersion among the three biological replicates, confirming the consistency of experimental operations across replicates (Figures 3A–E). Significant differences in crude starch, reducing sugar, and nitrogen content were observed among the three rice varieties (Figures 3A–C). At the early stage of fermentation, DJG exhibited the lowest crude starch and the highest reducing sugar levels, indicating more active saccharification and microbial utilization of sugars. The high sucrose content in DJG was rapidly hydrolyzed into reducing sugars, serving as substrates for subsequent fermentation. As fermentation progressed, reducing sugars were gradually consumed, and by the late stage, only nitrogen content remained significantly different among the groups.

Moisture and acidity showed no significant differences throughout the process (Figures 3D–E), suggesting comparable acid-producing and dominant microbial activity across varieties. Initial nitrogen levels mainly originated from protein degradation. After 1 day of saccharification, DJG, with the highest protein content, also showed the highest nitrogen level. This increase was likely due to the efficient utilization of amino acids, particularly arginine and glutamine, by microbes and enzymatic systems, along with enhanced protein degradation that accelerated early nitrogen release. During early fermentation, rapid microbial growth led to substantial nitrogen consumption, with the fastest depletion observed between days 0 and 4 (Figure 3C). In the later stages, the consumption rate slowed, and a temporary increase in nitrogen was noted, likely due to yeast and microbial autolysis releasing endogenous nitrogen. Throughout the process, DJG maintained the highest nitrogen content. Nitrogen availability strongly influences the formation of higher alcohols and branched-chain acids, which serve as key precursors for ester synthesis.

### 3.3 Microbial diversity of *Zaopei* based on metagenomic analysis

**Figure 4:**
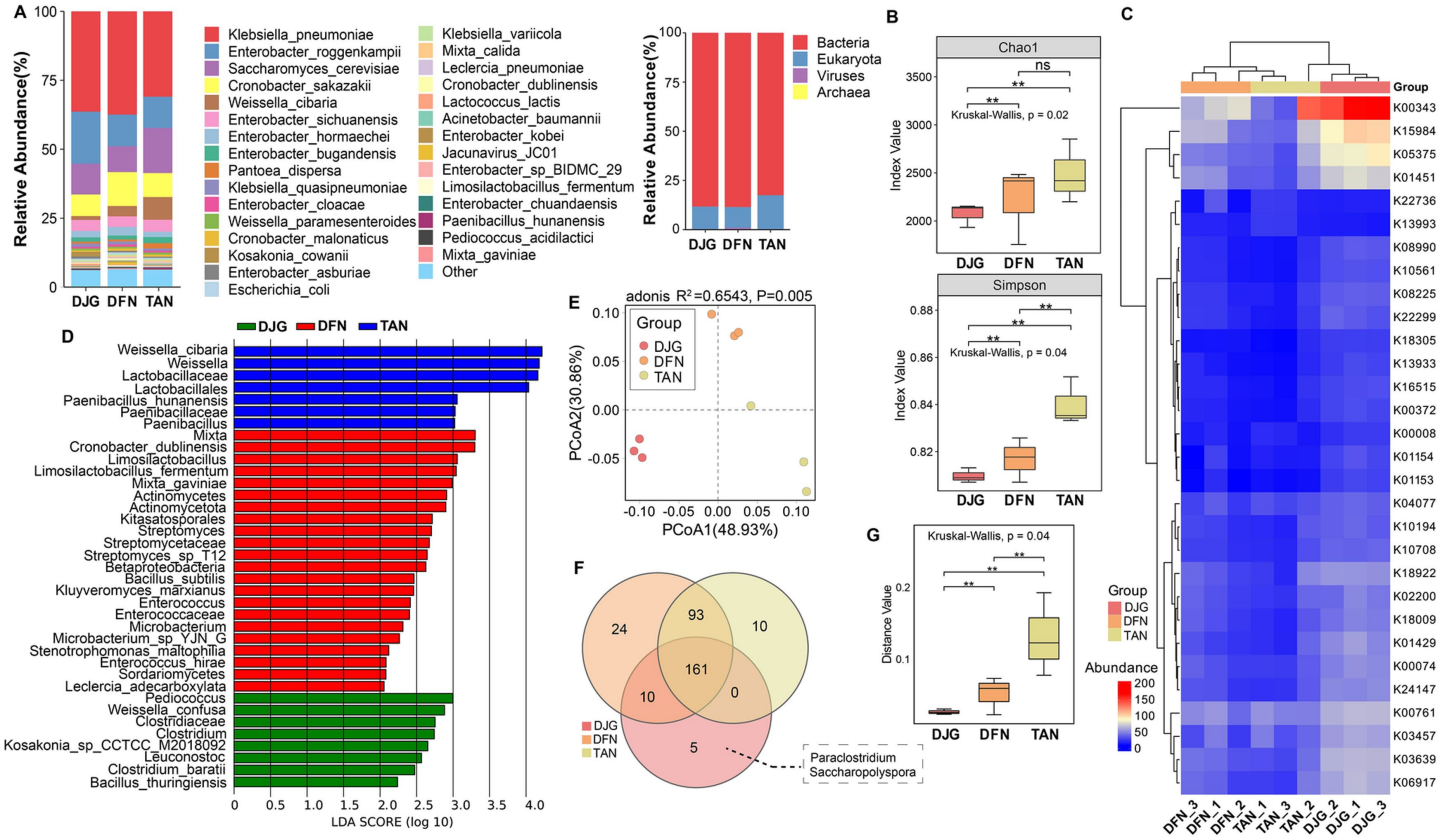
Metagenomic analysis of microbial communities in Zaopei. **(A)** Stacked bar chart of species-level relative abundances showing the top 30 most abundant taxa; others were grouped as “Other,” and kingdom-level classification. **(B)** Alpha diversity indices represent species richness and evenness. **(C)** Heatmap of significantly different functional categories. Non-parametric statistical tests (Wilcoxon/Kruskal–Wallis) were applied, and the top 30 categories with *p* < 0.05 were visualized. **(D)** LDA score plot based on LEfSe analysis, indicating taxa with significant differences among groups; the *x*-axis shows LDA scores, and the *y*-axis lists discriminatory taxa. **(E)** Principal component analysis (PCA) plot with ANOSIM test assessing group-level differences. **(F)** Venn diagram of differentially abundant species. **(G)** Boxplot of beta diversity illustrating within-group variability.

Alpha diversity differed significantly among the rice varieties. TAN samples exhibited the highest microbial evenness and community stability, followed by DFN, while DJG showed the lowest evenness, suggesting dominance by a few taxa (Figure 4B). Beta diversity also revealed significant differences in community composition among the three groups (Adonis analysis, *R*^2^ = 0.6543, *p* = 0.005; Figures 4E,G), indicating that glutinous rice variety strongly influences microbial community structure during *Xiaoqu* fermentation. At the species level, multiple differentially abundant microbes were detected across the three *Zaopei* groups, Bacteria were dominant (Figure 4A). *Klebsiella*, *Enterobacter*, *Saccharomyces*, *Cronobacter*, and *Weissella* were among the most abundant genera (Figure 4A; Supplementary Table S1). All samples showed high abundance of Enterobacteriaceae in the early fermentation stage, particularly in DJG. This family is a known contributor to histamine (HIS) formation in Chinese Huangjiu, including species such as *Enterobacter asburiae*, *Enterobacter cloacae*, and *Cronobacter sakazakii* (Zhang et al., 2018). At the same time, *Enterobacter* and *Klebsiella* possess strong carbohydrate metabolism capabilities and can utilize diverse carbon sources in brewing substrates, such as starch, pectin, inulin, and locust bean gum (Ochuba and von Riesen, 1980). These bacteria can survive in the early fermentation environment when pH is still high and ethanol levels are low. Both genera also produce volatile sulfur compounds and ketones, including diacetyl, which imparts buttery aromas. When coexisting with yeast during the early stage, they may compete for nitrogen and sugars, and in some cases, inhibit yeast activity (Virkajärvi et al., 2001).

LDA effect size (LEfSe) analysis revealed that TAN samples were enriched in lactic acid bacteria, including *Weissella* and *Lactobacillaceae*, as well as functional spore-forming bacteria such as *Paenibacillus*. Certain lactic acid bacteria are considered key contributors to lipid hydrolysis, and the higher fat content in TAN may have promoted their enrichment. DFN samples exhibited co-occurrence of lactic acid bacteria and actinomycetes, indicating a more complex community structure, with species such as *Kluyveromyces marxianus* and the functional strain *Cronobacter sakazakii* present. In DJG samples, which had the highest total amino acid and alanine contents in brown rice, post-fermentation *Zaopei* was enriched not only in *Saccharomyces cerevisiae* but also in *Pediococcus*, which expresses alanine dehydrogenase (AlaDH), and *Clostridium*, an anaerobe that utilizes amino acids as primary carbon sources (Sharma et al., 2021). *Clostridium* can rapidly proliferate under anaerobic conditions with sufficient glutamate and glutamine, showing strong competitiveness but often reducing community diversity (Laanbroek et al., 1979; Figure 4D). Two unique genera were identified in DJG: *Paraclostridium*, a subgroup of *Clostridium*, and *Saccharopolyspora*, which is known for erythromycin production and antimicrobial activity. Its glycosyltransferases DesVII/DesVIII exhibit high substrate flexibility and are involved in the biosynthesis of structurally diverse glycoside derivatives, which play a crucial role in the modification of aroma, bitterness, and sweetness precursors (Wu et al., 2016; Figure 4F). *Saccharopolyspora* is also essential for amino acid synthesis, fatty acid biosynthesis, triglyceride hydrolysis, and ester formation. Its high abundance in well-fermented Huangjiu has been linked to elevated nutritional value and characteristic flavor profiles (Liu et al., 2019). Although DJG exhibited the lowest microbial diversity, it showed significantly higher abundances of many functional genes compared to DFN and TAN. These included NADH dehydrogenase (K00343), 3-hydroxybutyryl-CoA dehydrogenase (K00074), and fructoselysine 6-phosphate deglycase (K10708), among others (Figure 4C; Supplementary Table S2), suggesting enhanced capacity for energy metabolism, nucleic acid biosynthesis, and environmental adaptation.

### 3.4 Metaproteomic profiling of *Zaopei* in the late fermentation stage

**Figure 5:**
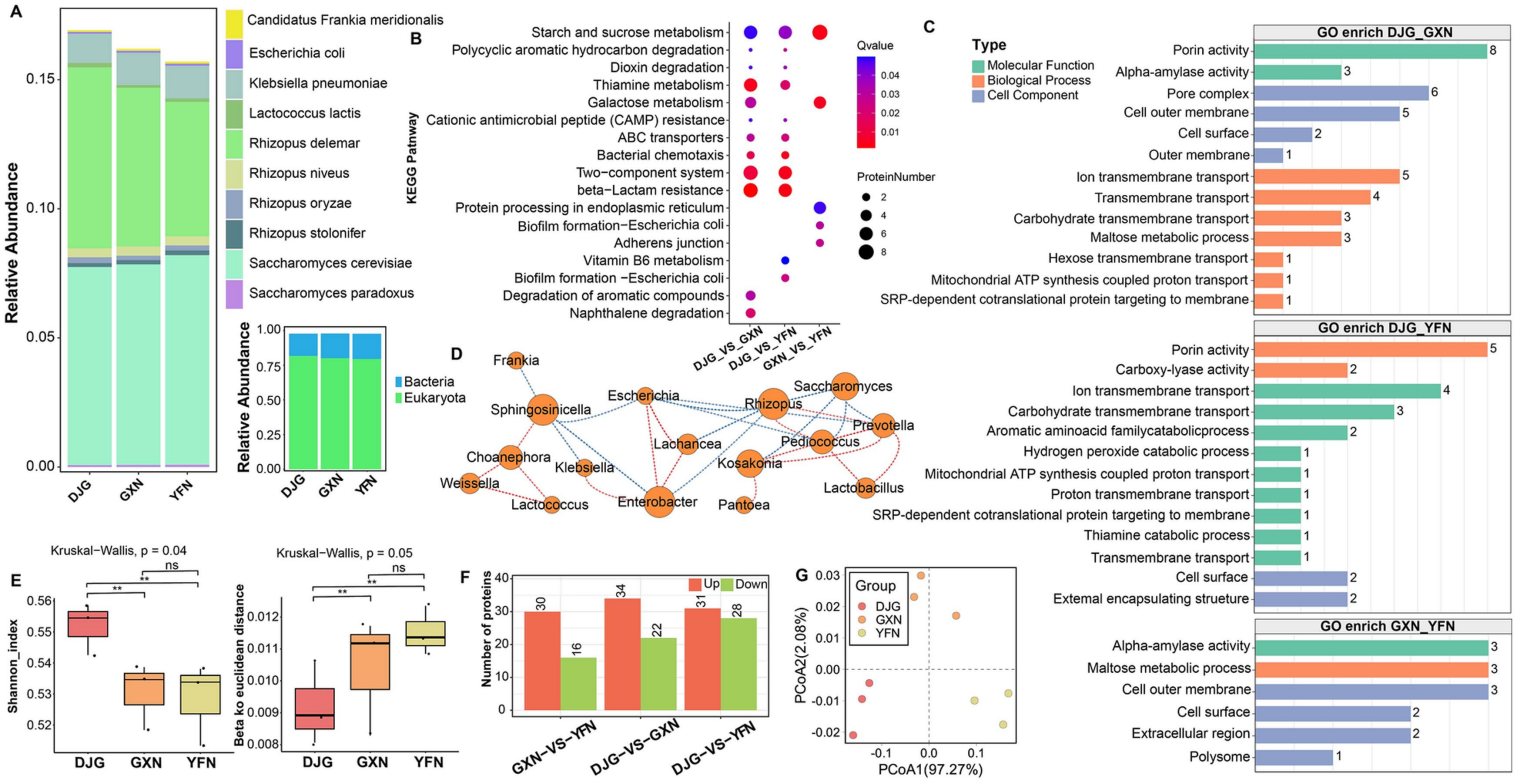
Differential metaproteomic analysis of *Zaopei* samples. **(A)** Taxonomic abundance at the species and kingdom levels. **(B)** KEGG pathway enrichment of differentially expressed proteins (bubble plot). **(C)** GO enrichment of differentially expressed proteins. **(D)** Microbial correlation network based on significant associations (*R* > 0.8, *p* < 0.05). **(E)** Alpha and beta diversity of microbial species. **(F)** Bar chart showing the number of differentially expressed proteins across groups. **(G)** Principal coordinates analysis (PCoA).

At the metaproteomic level, fungi were dominant. In contrast to the metagenomic results, this is a common phenomenon in solid-state fermentation. Liu et al. (2019) similarly observed in Huangjiu *Zaopei* that fungi dominated the functional proteins even when bacterial reads were higher in metagenomic data. Despite nitrogen depletion in the late fermentation stage, *Rhizopus* and *Saccharomyces* maintained high protein expression, likely via starvation-response mechanisms (Figure 5A). KEGG pathway enrichment indicated that DJG exhibited notable functional advantages, with differentially expressed proteins enriched in carbohydrate metabolism (starch and sucrose metabolism, thiamine metabolism), environmental adaptation (two-component systems, bacterial chemotaxis, ABC transporters), and stress resistance pathways (CAMP resistance, beta-lactam resistance; Figure 5B; Supplementary Table S3). GO enrichment analysis revealed that DJG-specific proteins were associated with core metabolic functions such as porin activity, *α*-amylase activity, transmembrane transport, aromatic amino acid degradation, and antioxidant response, reflecting efficient saccharification and substrate utilization (Figure 5C; Supplementary Table S4). Differential taxa formed a core microbial interaction network centered on *Enterobacter*, *Rhizopus*, and *Saccharomyces*. Although *Sphingosinicella* was not a dominant fermentative genus, its correlations with key microbes may result from ecological or nutritional interactions. As fermentation progressed, low-abundance genera such as *Pseudomonas*, *Ralstonia*, and *Sphingomonas* were detected (Figure 5D; Supplementary Table S5), often considered “satellite” or “non-core” microbes. Previous studies have reported that non-dominant taxa in traditional fermentations (e.g., Baijiu, Huangjiu, black glutinous rice wine) frequently coexist with core flavor-producing microbes and contribute to microbial interactions (Zou et al., 2023). DJG samples showed the highest Shannon diversity in protein expression and the lowest inter-sample variability (Figure 5E), with the greatest number of differentially expressed proteins compared to other varieties (Figure 5F). These results suggest that although fewer taxa dominated DJG, its microbial community has higher metabolic activity and protein expression capacity, providing mechanistic insight into its superiority as a high-quality brewing glutinous rice variety.

### 3.5 Untargeted LC–MS metabolomic analysis of *Zaopei*

**Figure 6:**
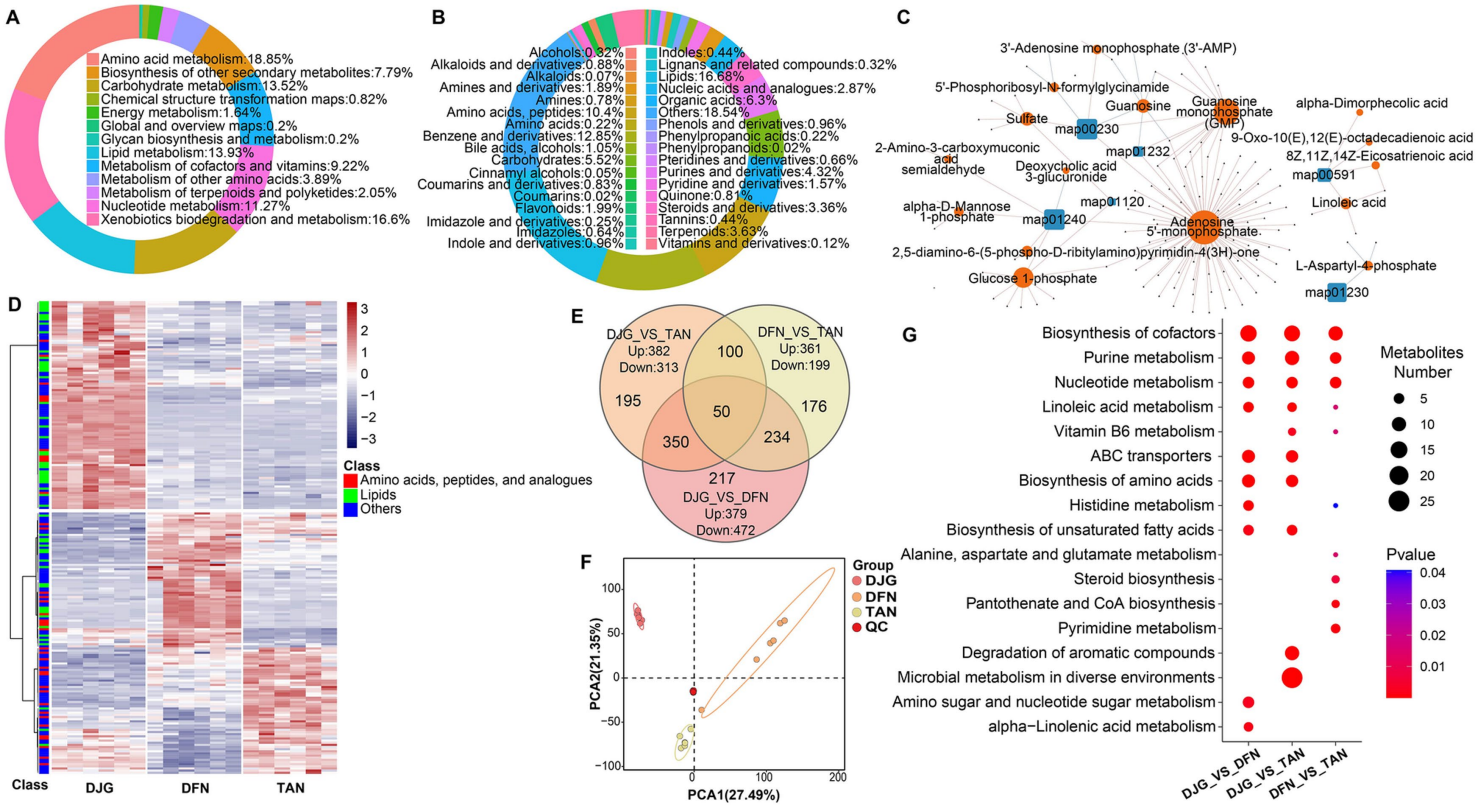
Untargeted LC–MS-based metabolomic analysis of *Zaopei*. **(A,B)** Classification of metabolic pathways and corresponding metabolite counts. Colors indicate different pathway or metabolite categories. **(C)** KEGG enrichment network of differential metabolites in DJG compared with the other two groups. Blue squares represent metabolic pathways; orange-red circles indicate enriched metabolites within each pathway; black dots represent enzymes associated with the corresponding metabolites. **(D)** Hierarchical clustering of differential metabolite expression across samples. Each row represents one metabolite and each column one sample. Color gradient from red to blue indicates increasing expression levels. **(E)** Venn diagram showing the number of differential metabolites among the three groups. **(F)** PCA of metabolite profiles across samples. **(G)** Bubble plot of KEGG pathway enrichment for differential metabolites in each group.

The metabolites detected in *Zaopei* were mainly involved in amino acid biosynthesis (18.85%), xenobiotic degradation (16.6%), lipid metabolism (13.93%), and carbohydrate metabolism (13.52%) (Figure 6A). Lipids (16.68%) and amino acids/peptides (10.4%) were the most abundant metabolite classes. In DJG samples, 40 lipids were upregulated and 16 downregulated, with upregulated compounds primarily consisting of short-chain fatty acids, linoleic acid, palmitoleic acid, stearic acid, and their derivatives (Supplementary Table S6). Given that DJG had the lowest initial fatty acid and total lipid content among the varieties, the fatty acids accumulated in late-stage *Zaopei* likely originated from microbial *de novo* synthesis via acetyl-CoA and from microbial autolysis under carbon and nitrogen limitation (Figure 2). Most amino acid-related compounds in *Zaopei* were dipeptides formed as intermediates of protein degradation. For example, *γ*-glutamylphenylalanine, synthesized from glutamate and phenylalanine, is a known umami-enhancing compound. It was significantly elevated in TAN and lowest in DJG, likely due to the high abundance of *Lactobacillus* in TAN. Species such as *Saccharomyces*, *Bacillus*, *Rhizopus*, and *Pediococcus* have been positively correlated with levels of bitter and umami amino acids (Liang et al., 2020). Most amino acid derivatives downregulated in DJG were N-acyl amino acids. Members of the *Clostridium* genus are capable of degrading branched-chain acylated derivatives such as isovalerylglutamic acid into short-chain fatty acids like isovaleric acid and glutamate (Yang et al., 2024).

Functional compounds such as phenolics, alkaloids, and terpenoids (Figure 6B) enhanced both the flavor complexity and potential health benefits of Baijiu. In DJG *Zaopei*, 4-Hydroxycinnamic acid was significantly upregulated, whereas ferulic acid was downregulated. Previous studies have shown that phenolic compounds like 4-Hydroxycinnamic and ferulic acids primarily originate from raw materials rather than microbial metabolism (Liu et al., 2019). Among the three glutinous rice varieties, DJG contained the highest total phenolic content (4.35 mg/g), while TAN had the lowest (3.33 mg/g) (Figure 1D). Correspondingly, the Baijiu derived from DJG exhibited increased levels of volatile phenolic compounds, including 4-vinylphenol, 2-methoxy-4-vinylphenol, 4-ethyl-2-methoxyphenol, and 2-methoxyphenol—most of which are downstream metabolites of ferulic acid. This likely explains the observed decrease in ferulic acid during fermentation (Figure 2). Previous research by our group showed that DJG tended to accumulate a thicker cuticle layer at 21 days after endosperm development, compared to other varieties. The cuticle, composed primarily of lipids (30–50%) and phenolics (20–40%), serves as a major source of phenolics (~20%) and a minor source of lipids (<5%, with 65–75% of lipids originating from the embryo) (Juliano and Tuaño, 2019). Approximately 30% of phenolics bound to cutin or fatty acid chains via ester linkages are released during steaming, with the remainder released through microbial enzymatic hydrolysis (Philippe et al., 2020). These released phenolic acids are then decarboxylated by phenolic acid decarboxylase (PAD) during fermentation, forming volatile phenolic compounds such as 4-vinylphenol.

Pathway network analysis revealed that differential metabolites in DJG were primarily enriched in energy metabolism, carbohydrate metabolism, and fatty acid metabolism. AMP, glucose-1-phosphate, and linoleic acid were among the most upregulated metabolites in DJG (Figure 6C; Supplementary Table S6), indicating that the fermentation system remained in a high-energy-demand phase, characterized by active glycolysis and biosynthetic reactions. A portion of the AMP may have originated from microbial autolysis. Cluster heatmaps and PCA plots confirmed good within-group reproducibility. Besides the notable upregulation of amino acids and lipids in DJG, other differential modules showed no clear pattern of metabolite classification (Figures 6D,F). The Venn diagram showed that, in pairwise comparisons, DJG exhibited a greater number of intergroup differences than the other two groups (Figure 6E). KEGG enrichment analysis further demonstrated significant accumulation of metabolites involved in nucleotide metabolism, amino acid biosynthesis, fatty acid metabolism, and coenzyme biosynthesis pathways in DJG samples (Figure 6G). These pathways are closely associated with efficient energy utilization, active enzymatic systems, and enhanced flavor formation. The results are consistent with both metagenomic and metaproteomic findings, providing molecular evidence for the superior fermentation performance of the DJG variety.

### 3.6 Differential profiles of volatile flavor metabolites in Baijiu

**Figure 7:**
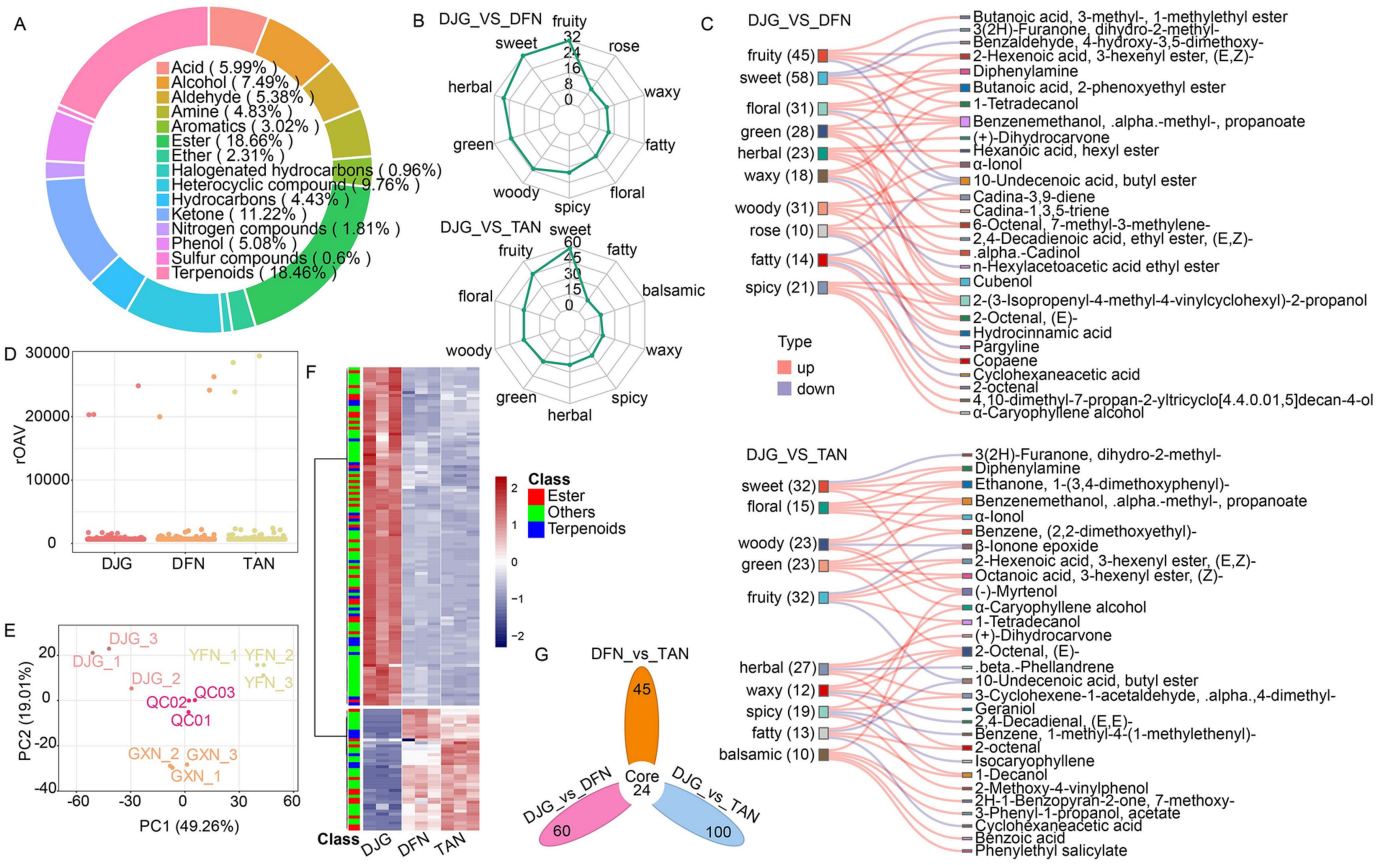
Metabolomic analysis of Baijiu volatile compounds. **(A)** Circular plot showing the number and classification of all detected volatile metabolites in Baijiu. **(B)** Radar chart comparing sensory attributes of DJG with the other two groups, based on the top 10 sensory descriptors with the most annotated metabolites. **(C)** Flavoromics Sankey diagram: left column represents sensory attributes, right column represents differential metabolites. Red lines indicate upregulated metabolites; blue lines indicate downregulated metabolites. For each flavor, the five metabolites with the highest fold change are shown. **(D)** Relative odor activity values (rOAV) of volatile compounds. **(E)** PCA plot showing sample clustering across groups and pooled QC samples. **(F)** Hierarchical clustering heatmap of differential volatile metabolites; red to blue indicates high to low abundance. **(G)** Venn diagrams displaying unique and shared differential volatile metabolites between pairwise group comparisons.

Metabolomic analysis of Baijiu revealed that DJG-derived samples exhibited superior flavor composition and sensory attributes compared to DFN and TAN. This was primarily characterized by a high enrichment of esters (18.66%) and terpenoids (18.46%) (Figure 7A), contributing to pronounced fruity, sweet, and floral notes. Clear distinctions in VOC profiles were observed between DJG and the other two groups (Figure 7G). Radar plots further confirmed that DJG liquor had significantly higher intensities in fruity, sweet, and floral aroma dimensions (Figure 7B). PCA analysis showed significant differences among the groups (Figure 7E). Esters and terpenoids were significantly upregulated in DJG (Figure 7F), while compounds associated with grassy and nutty aromas were relatively less abundant, resulting in a more rounded and mellow flavor profile. Most of the top-ranked aroma-contributing compounds were typical aromatic metabolites, such as acetate esters and phenylethanol (Figure 7C). Some aroma compounds, including nonanal and 1-octen-3-ol, may originate directly from cooked glutinous rice (Supplementary Table S7; Jidapa and Siree, 2008). These findings further support the favorable flavor potential of the DJG variety and its associated fermentation ecology in Baijiu production.

#### 3.6.1 Ester-derived flavor compounds

Esters represented the most abundant class of volatile compounds in the Baijiu, with the majority showing upregulation in DJG samples. The enriched esters were primarily fruity acetate esters and medium- to short-chain fatty acid esters, along with a smaller proportion of long-chain fatty acid esters and aromatic esters. A few acetate esters and medium/long-chain fatty acid esters were downregulated. DJG samples contained higher levels of acetate esters such as ethyl nonanoate, ethyl benzoate, and ethyl tridecanoate, which are known to impart floral and fruity notes (Xu et al., 2022). Alcohol acetyltransferase (AATase) preferentially catalyzes esterification between higher alcohols—produced via the Ehrlich pathway, such as isoamyl alcohol—and short-chain fatty acids. In contrast, despite the presence of N-acetyl-L-leucine in *Zaopei*, AATase exhibits low affinity toward this compound, resulting in the downregulation of DL-leucine, N-acetyl-, ethyl ester. Unsaturated fatty acids present in the bran layer can inhibit AATase activity and reduce acetate ester formation in fermented liquors (Ashida et al., 1987). There are two types of AATase: AATase I is sensitive to inhibition by unsaturated fatty acids, while AATase II is not. However, AATase I has broader substrate specificity and accounts for 70–80% of total esterification activity. In addition to inhibition by unsaturated fatty acids, anaerobic conditions combined with sufficient nitrogen can enhance the enzymatic activity of AATase I (Yoshimoto and Bogaki, 2023).

Alanine can be oxidized to pyruvate via alanine dehydrogenase (AlaDH), which enters the tricarboxylic acid (TCA) cycle during early fermentation when oxygen is still present, thereby releasing substantial energy. Arginine content showed a positive correlation with *Pediococcus*, which utilizes arginine efficiently through the arginine deiminase (ADI) pathway to produce citrulline (Liang et al., 2020). In DJG samples, which had the highest arginine content among the three rice varieties, citrulline was the most upregulated amino acid derivative in the post-fermentation *Zaopei* (Figure 2; Supplementary Table S6). Citrulline is a known precursor for ethyl carbamate (EC) and also serves as a major energy source. In anaerobic conditions, *Clostridium* species preferentially metabolize citrulline for energy production rather than EC formation. For example, in *Clostridium carboxidivorans* P7, the addition of citrulline increased ATP levels by 3.4-fold and ethanol production by 20% (Li et al., 2021). Moreover, urea—another key EC precursor—was not detected in *Zaopei*, suggesting that citrulline in DJG samples was mainly derived from the ADI pathway rather than the ornithine cycle. In addition to EC, the volatile compound dimethyl carbamate was also detected in the liquor, which can be formed by the reaction between amines and carbamoyl precursors. Biogenic amines, derived from amino acid decarboxylation, are known to correlate positively with arginine levels in raw materials and putrescine concentrations in Huangjiu (Zhao et al., 2024). Although biogenic amines do not carry over into distilled Baijiu, the higher amine content in DJG *Zaopei* likely contributed to the elevated levels of dimethyl carbamate (Figure 2).

#### 3.6.2 Differential alcohol-derived flavor compounds

Ethanol concentrations showed no significant differences among the samples. Three thiol compounds—1-dodecanethiol, 2-thiophenemethanethiol, and benzenethiol—were significantly downregulated in DJG. In contrast, 1-hexanol, the most abundant alcohol among the differential metabolites, was significantly upregulated in DJG. This compound is a key aroma-active component in strong-aroma Baijiu, contributing green, fruity, fatty notes as well as bitterness. 1-Hexanol is typically produced as a metabolic byproduct of *Clostridium* or derived from the oxidative degradation of linoleic acid. Although methionine levels—an important sulfur-containing amino acid—were higher in DJG rice, the difference was not statistically significant; cysteine was not detected. Besides *Saccharomyces* and *Lactobacillus*, which are known to produce thiols and sulfide, the dominant *Clostridium* may convert methionine into methanethiol, ammonia, and *α*-ketobutyrate via *γ*-lyase activity (Landaud et al., 2008). Despite lower levels of free thiols such as 1-dodecanethiol in DJG, a significant increase in dimethyl trisulfide (DMTS) was observed, suggesting that thiols were oxidized into polysulfides during distillation. In addition, aldehydes generated from fatty acid oxidation in DJG *Zaopei* may react with thiols to form thioethers such as 1-propenyl propyl ether, which also promote DMTS formation (Belda et al., 2017). The DMTS concentration in DJG liquor was 0.99 μg/L (Supplementary Table S7), well below the sensory threshold for off-odor (5 μg/L), and is considered beneficial for enhancing the flavor complexity of strong- and sauce-aroma Baijiu (Table 1).

**Table 1:** Significant differential VOCs (rOAV > 1) between DJG and other groups.

Compounds	Odor	rOAV (DJG)	rOAV (DFN)	rOAV (TAN)	Log2FC(DJG-vs-DFN)	Log2FC(DJG-vs-TAN)
2-Octenal, (E)-	Fresh, cucumber, fatty, green, herbal, banana, waxy, leafy	2.77	0.62	0.69	2.33	2.28
2,4-Decadienal, (E, E)-	Dusty, waxy, oily, soapy	5.44	12.78	17.42	−1.15	−1.48
Ethanone, 1-(2-aminophenyl)-	Grape, sweet	129.20	47.59	36.11	1.58	2.10
1-Hexanol	Ethereal, fusel, oily, fruity, alcoholic, sweet, green	7.48	2.67	3.06	1.60	1.50
2-Methoxy-4-vinylphenol	Spicy, raisin	16.14	6.03	4.67	1.57	2.06
2-Octenal	Fatty, green, herbal	41.62	9.36	10.41	2.33	2.28
Dimethyl trisulfide	Sulfury, cooked onion, savory, meaty	124.35	5.51	3.13	4.67	5.61
3(2H)-Furanone, dihydro-2-methyl-	Sweet, solvent, bread, buttery, nutty	8.61	337.25	1344.49	−5.18	−7.03
Nonanoic acid, ethyl ester	Fruity, rose, waxy, rummy, wine, natural, tropical	1.45	0.47	0.77	1.83	1.21

#### 3.6.3 Differential aldehyde compounds

Most of the differential aldehydes were derived from linoleic acid degradation. Linoleic acid undergoes autoxidation to form 9-ROOH, which is cleaved via *β*-scission to generate 2,4-decadienal. This compound can be further oxidized into crotonaldehyde and subsequently react with 1-heptene to form 2-octenal. In DJG samples, lower levels of 2,4-decadienal and higher levels of 2-octenal were detected, suggesting a more oxidative environment in DJG *Zaopei* during the late fermentation phase. Both compounds contribute to fatty and green aromas (Table 1). 2,4-Decadienal has been identified as a key aroma compound in Qingya-flavored Baijiu brewed with glutinous rice (Wu et al., 2024). Similarly, (E)-2-octenal and 2,4-heptadienal have been reported as essential contributors to the fresh fruity aroma of yellow rice wine fermented with glutinous and black rice (Tang and Peng, 2024). Due to their low boiling points and high volatility, aldehydes not only contribute directly to aroma but also facilitate the release of other volatile compounds, thereby enhancing the overall flavor profile. Their concentrations are closely linked to the raw materials used in fermentation (Wei et al., 2020).

#### 3.6.4 Other differential flavor compounds

Terpenes were predominantly upregulated in DJG, including monoterpenes such as myrtenol and geraniol, which contribute floral, fruity, sweet, and rose-like aromas. Additionally, several polycyclic sesquiterpenes and diterpenoids with herbal, woody, and spicy notes were identified. Only a few terpenes showed significant differences in *Zaopei*, likely because they exist in bound forms and are mainly released during distillation, thereby amplifying differences in the final liquor. Hydrocarbons, primarily straight-chain alkenes and alkanes, are derived either from the reduction of long-chain fatty acids (e.g., stearic and palmitic acids) during distillation or through decarboxylation and dehydrogenation of unsaturated fatty acids such as oleic acid. As DJG *Zaopei* contained higher levels of C18 fatty acids, corresponding hydrocarbons were also upregulated in DJG liquor. Although hydrocarbons have limited aroma, they assist in stabilizing volatile compounds such as esters and terpenes by adsorption. Ketones, including aliphatic, cyclic, aromatic, and heterocyclic types, also displayed a pattern similar to that of hydrocarbons. All except heterocyclic ketones were elevated in DJG samples. These compounds are indicative of fatty acid oxidation and phenolic acid degradation. Additionally, several heterocyclic compounds such as pyrazines, thiophenes, and furans were significantly upregulated in DJG liquor, contributing herbal, woody, sweet, and caramel-like aromas. Among Ehrlich pathway-derived acids, divergent trends were observed: 3,3-dimethylbutanoic acid increased, while 2-methylbutanoic acid decreased, suggesting different microbial utilization efficiencies for leucine and isoleucine. Elevated levels of syringic acid further confirmed active degradation of phenolic precursors like ferulic acid in *Zaopei*.

### 3.7 OVA analysis of differential volatile metabolites in Baijiu

Over 2,000 aroma compounds have been identified in Baijiu, but only a limited number of key volatiles play a decisive role in defining sensory characteristics across different Baijiu types. Since concentration alone does not fully explain perceptual differences, compounds with an odor value (OVA) greater than 1 are generally considered to contribute more directly to aroma perception. In this study, 66 differential aroma compounds with OVA > 1 were identified (Figure 7D), including ethyl decanoate, isoamyl acetate, phenylethanol, ethyl octanoate, and ethyl laurate (Supplementary Table S8). Among them, nine VOCs showed significant concentration differences between DJG and the other two groups (Table 1). The compound with the highest OVA was 2(5H)-Furanone, 5-ethyl-3-hydroxy-4-methyl- (Figure 7D), though its concentration was very low and did not differ significantly among groups. This compound is synthesized via a condensation reaction between *α*-ketobutyrate (derived from threonine deamination) and propanal (Piornos et al., 2020). No threonine or its derivatives were detected among differential metabolites in *Zaopei*, suggesting that amino acid metabolism did not shift globally, but rather targeted specific precursors and derivatives. Another furanone with high OVA, 3(2H)-Furanone, dihydro-2-methyl-, was significantly downregulated in DJG samples. This compound is formed non-enzymatically from small molecules such as pyruvate or lactic acid under reductive and acidic conditions. In addition to furanones, other key differential aroma compounds included dimethyl trisulfide, 2-octenal, and 2,4-decadienal (E, E), all of which contribute distinct sensory characteristics to the final liquor.

### 3.8 The relationship between microbial diversity and functionality

In this study, DJG exhibited the lowest microbial diversity but the highest metabolic activity across metagenomic, metaproteomic, and metabolomic levels. Despite reduced taxonomic complexity, DJG was dominated by functionally specialized taxa, including *Saccharomyces*, *Pediococcus*, *Clostridium*, and *Saccharopolyspora*, which are associated with enhanced pathways for carbohydrate metabolism, amino acid turnover, and fatty acid synthesis. This functional specialization directly shapes flavor outcomes. For instance, *Saccharomyces* and *Pediococcus* in DJG drove elevated production of short and medium chain fatty acids and their corresponding esters, such as ethyl acetate, ethyl laurate, and ethyl nonanoate, contributing fruity and floral notes in the final liquor. Meanwhile, the dominance of *Clostridium* facilitated amino acid catabolism, increasing alanine and arginine derived metabolites and promoting the formation of sulfur-containing volatiles such as dimethyl trisulfide, which heightens flavor complexity without exceeding sensory thresholds. Additionally, higher phenolic precursor levels from DJG rice resulted in elevated volatile phenolics including 4-vinylphenol, contributing smoky, spicy, and toasted aromas in the final Baijiu. From an industrial perspective, this streamlined microbial consortium may enhance flavor consistency because key metabolic functions are concentrated in a small number of stable core taxa rather than distributed across diverse microbial groups (Louca et al., 2018). Therefore, fluctuations in minor taxa have less impact on final flavor profiles, allowing predictable ester formation, phenolic conversion, and amino acid utilization across batches. Such properties are advantageous for large-scale production where reproducible sensory quality is required. However, the low microbial diversity also reduces functional redundancy; if dominant taxa such as *Clostridium* or *Saccharomyces* are inhibited due to raw material variability or environmental stress during scale up, functional pathways (e.g., sulfur metabolism, saccharification, amino acid catabolism) may collapse, leading to off-flavors or insufficient ester formation (Louw et al., 2023). In contrast, TAN and DFN possess higher diversity and microbial co-occurrence networks, enabling broader metabolic buffering capacity but also contributing to greater flavor variability between batches.

Thus, DJG represents a high-efficiency but low redundancy fermentation system, which is optimal for standardized production under controlled industrial conditions, but requires stricter environmental management to maintain stability during scale up. This is a necessary consideration for practical industrial production.

### 3.9 Effects of glutinous rice vs. other grains on fermentation microbiota and flavor

When compared with previous studies on non-glutinous rice and other cereals, our findings highlighted both shared and distinct mechanisms by which grain composition shapes fermentation microbiota and flavor formation. In sorghum-based strong-flavor Baijiu, variation in tannin content has been shown to restructure microbial communities and drive divergent ester profiles, with high-tannin sorghum enriching specific anaerobes and correlating with increased ester formation (Xu et al., 2018; Liu et al., 2023). Similarly, in Huangjiu and rice wine systems, the use of alternative cereals such as buckwheat, black rice, and Chinese wild rice has been reported to enhance amino acid- and phenolic-derived aroma complexity, often at the cost of ethanol yield (Yang et al., 2020; Shen et al., 2024; Zhao et al., 2024; Tang and Peng, 2024). These studies collectively emphasize that non-starch components—particularly proteins, lipids, tannins, and phenolics—are key levers for modulating microbial succession and volatile formation beyond the effects of starch structure and amylose content.

Compared with other grains and non-glutinous rice, glutinous rice improves alcohol yield because of its high amylopectin content, while its favorable gelatinization properties facilitate microbial enzymatic utilization, and its higher levels of amino acids and phenolic compounds enrich fermentation derived flavor (Figures 1G–J). These attributes are particularly pronounced in DJG, which contained high sucrose and amino acid contents and relatively low initial fatty acid levels, resulting in a low diversity dominance driven microbial community where metabolic functions are primarily contributed by *Saccharomyces*, *Pediococcus*, *Clostridium*, and *Saccharopolyspora*, thereby enhancing pathways associated with carbohydrate utilization, amino acid catabolism, and fatty acid metabolism. This translated into selective enrichment of fruity and floral esters, sulfur compounds such as dimethyl trisulfide, and volatile phenolics including 4-vinylphenol and 2-methoxy-4-vinylphenol in the final Baijiu, yielding a rounded, sweet–fruity–floral profile distinct from the grassy or nutty notes more common in non-glutinous or high-tannin grain fermentations (Wu et al., 2024; Xu et al., 2022; Tang and Peng, 2024).

## 4 Conclusion

This study systematically investigated the impact of different glutinous rice varieties on Baijiu fermentation. Unsaturated fatty acids in glutinous rice were found to inhibit microbial proliferation during early fermentation and suppress AATase activity, limiting acetate ester formation. During microbial growth, unsaturated fatty acids are synthesized for membrane construction and later released through autolysis to serve as precursors for esters, aldehydes, hydrocarbons, and ketones. Among available sugars, sucrose is more readily utilized by microbes. Genera such as *Klebsiella* and *Enterobacter* can accelerate early-stage sugar metabolism, promoting rapid microbial proliferation. Although *Enterobacter* produces biogenic amines, these compounds are not retained in distilled Baijiu and pose no risk. The amino acid composition of the raw grain plays a key role in shaping microbial diversity. Alanine and arginine promote the enrichment of *Pediococcus* and *Clostridium*. *Pediococcus* degrades arginine via the ADI pathway to produce citrulline, which is further utilized by *Clostridium* for ATP generation, supporting metabolic activity. *Clostridium* also contributes to the conversion of methionine into dimethyl trisulfide, enhancing aroma complexity.

Volatile phenolic compounds in the final liquor are derived from raw material precursors such as 4-Hydroxycinnamic acid and ferulic acid. Overall, this study demonstrates that compositional differences among glutinous rice varieties significantly influence microbial diversity, especially during the early fermentation phase, offering insights for the selection and breeding of rice varieties tailored for Baijiu production.

## Data Availability

The datasets presented in this study can be found in online repositories. The names of the repository/repositories and accession number(s) can be found in the article/Supplementary material.

## Footnotes

1
https://www.thegpm.org/TANDEM/

2
http://www.maxquant.org

## References

[ref1] Andersson U. Rådström P. (2002). Physiological function of the maltose operon regulator, MalR, in *Lactococcus lactis*. BMC Microbiol. 2:28. doi: 10.1186/1471-2180-2-28, 12296976 PMC130022

[ref2] Arik Kibar E. A. Gönenç İ. Us F. (2014). Effects of fatty acid addition on the physicochemical properties of corn starch. Int. J. Food Prop. 17, 204–218. doi: 10.1080/10942912.2011.619289

[ref3] Ashida S. Ichikawa E. Suginami K. Imayasu S. (1987). Isolation and application of mutants producing sufficient isoamyl acetate, a sake flavor component. Agric. Biol. Chem. 51, 2061–2065. doi: 10.1080/00021369.1987.10868367

[ref4] Belda I. Ruiz J. Esteban-Fernández A. Navascués E. Marquina D. Santos A. . (2017). Microbial contribution to wine aroma and its intended use for wine quality improvement. Molecules 22:189. doi: 10.3390/molecules22020189, 28125039 PMC6155689

[ref5] Chen C. Liu Y. Tian H. Ai L. Yu H. (2020). Metagenomic analysis reveals the impact of JIUYAO microbial diversity on fermentation and the volatile profile of Shaoxing-jiu. Food Microbiol. 86:103326. doi: 10.1016/j.fm.2019.103326, 31703871

[ref6] Hu X. Fang C. Zhang W. Lu L. Guo Z. Li S. . (2023). Change in volatiles, soluble sugars and fatty acids of glutinous rice, japonica rice and indica rice during storage. LWT 174:114416. doi: 10.1016/j.lwt.2022.114416

[ref7] Jiang S. Huang W. Rong Y. Tao Y. Tang Y. (2002). Study on bittering mechanism of sweeten glutinous rice wine. Liquor Making 29, 43–45.

[ref8] Jidapa A. Siree C. (2008). Comparative study on aroma-active compounds in Thai, black and white glutinous rice varieties. Agric. Nat. Resour. 42, 715–722.

[ref9] Juliano B. O. Tuaño A. P. P. (2019). Gross structure and composition of the rice grain. Rice 4, 31–53. doi: 10.1016/B978-0-12-811508-4.00002-2

[ref10] Kang B. S. Lee J. E. Park H. J. (2014). Qualitative and quantitative prediction of volatile compounds from initial amino acid profiles in Korean rice wine (makgeolli) model. J. Food Sci. 79, C1106–C1116. doi: 10.1111/1750-3841.12489, 24888253

[ref11] Laanbroek H. J. Smit A. J. Nulend G. K. Veldkamp H. (1979). Competition for L-glutamate between specialised and versatile Clostridium species. Arch. Microbiol. 120, 61–66. doi: 10.1007/BF00413275, 426599

[ref12] Landaud S. Helinck S. Bonnarme P. (2008). Formation of volatile sulfur compounds and metabolism of methionine and other sulfur compounds in fermented food. Appl. Microbiol. Biotechnol. 77, 1191–1205. doi: 10.1007/s00253-007-1288-y, 18064452

[ref13] Li X. Han R. Bao T. Osire T. Zhang X. Xu M. . (2021). Citrulline deiminase pathway provides ATP and boosts growth of *Clostridium carboxidivorans* P7. Biotechnol. Biofuels 14:204. doi: 10.1186/s13068-021-02051-4, 34656154 PMC8520249

[ref14] Li S. Yuan R. Chen L. Wang L. Hao X. Wang L. . (2015). Systematic qualitative and quantitative assessment of fatty acids in the seeds of 60 tree peony (Paeonia section Moutan DC.) cultivars by GC–MS. Food Chem. 173, 133–140. doi: 10.1016/j.foodchem.2014.10.017, 25466004

[ref15] Liang Z. Lin X. He Z. Su H. Li W. Ren X. (2020). Amino acid and microbial community dynamics during the fermentation of Hong Qu glutinous rice wine. Food Microbiol. 90:103467. doi: 10.1016/j.fm.2020.103467, 32336361

[ref16] Liu S. Chen Q. Zou H. Yu Y. Zhou Z. Mao J. . (2019). A metagenomic analysis of the relationship between microorganisms and flavor development in Shaoxing mechanized huangjiu fermentation mashes. Int. J. Food Microbiol. 303, 9–18. doi: 10.1016/j.ijfoodmicro.2019.05.001, 31102963

[ref17] Liu M. Tang Y. Liu C. Tian X. Zhang J. Fan X. . (2023). Variation in microbiological heterogeneity in Chinese strong-flavor baijiu fermentation for four representative varieties of sorghum. Int. J. Food Microbiol. 397:110212. doi: 10.1016/j.ijfoodmicro.2023.110212, 37084618

[ref18] Louca S. Polz M. F. Mazel F. Albright M. B. N. Huber J. A. O’Connor M. I. . (2018). Function and functional redundancy in microbial systems. Nat. Ecol. Evol. 2, 936–943. doi: 10.1038/s41559-018-0519-1 29662222

[ref19] Louw N. L. Lele K. Ye R. Edwards C. B. Wolfe B. E. (2023). Microbiome assembly in fermented foods. Ann. Rev. Microbiol. 77, 381–402. doi: 10.1146/annurev-micro-032521-041956, 37713453

[ref20] Obukhova E. S. Murzina S. A. (2024). Mechanisms of the antimicrobial action of fatty acids: a review. Appl. Biochem. Microbiol. 60, 1035–1043. doi: 10.1134/S0003683824605158

[ref21] Ochuba G. U. Von Riesen V. L. (1980). Fermentation of polysaccharides by Klebsielleae and other facultative bacilli. Appl. Environ. Microbiol. 39, 988–992. doi: 10.1128/aem.39.5.988-992.1980, 7396489 PMC291464

[ref22] Park J. Park H. Y. Chung H. J. Oh S. K. (2023). Starch structure of raw materials with different amylose contents and the brewing quality characteristics of Korean rice beer. Foods 12:2544. doi: 10.3390/foods12132544, 37444283 PMC10341284

[ref23] Philippe G. Sørensen I. Jiao C. Sun X. Fei Z. Domozych D. S. . (2020). Cutin and suberin: assembly and origins of specialized lipidic cell wall scaffolds. Curr. Opin. Plant Biol. 55, 11–20. doi: 10.1016/j.pbi.2020.01.008, 32203682

[ref24] Pineda A. Carrasco J. Peña-Farfal C. Henríquez-Aedo K. Aranda M. (2012). Preliminary evaluation of biogenic amines content in Chilean young varietal wines by HPLC. Food Control 23, 251–257. doi: 10.1016/j.foodcont.2011.07.025

[ref25] Piornos J. A. Balagiannis D. P. Methven L. Koussissi E. Brouwer E. Parker J. K. (2020). Elucidating the odor-active aroma compounds in alcohol-free beer and their contribution to the Worty flavor. J. Agric. Food Chem. 68, 10088–10096. doi: 10.1021/acs.jafc.0c03902, 32799537 PMC7499417

[ref26] Sharma A. Noda M. Sugiyama M. Kumar B. Kaur B. (2021). Application of *Pediococcus acidilactici* BD16 (alaD +) expressing L-alanine dehydrogenase enzyme as a starter culture candidate for secondary wine fermentation. Biotechnol. Biotechnol. Equip. 35, 1643–1661. doi: 10.1080/13102818.2021.1995496

[ref27] Shen C. Yu Y. Zhang X. Zhang H. Chu M. Yuan B. . (2024). The dynamic of physicochemical properties, volatile compounds and microbial community during the fermentation of Chinese rice wine with diverse cereals. Food Res. Int. 198:115319. doi: 10.1016/j.foodres.2024.115319, 39643362

[ref28] Tang A. Peng B. (2024). Uncovering the flavor differences between black rice wine and glutinous rice wine by GC-MS, GC-IMS, HPLC, and electronic sensory analysis. Food Biosci. 60:104235. doi: 10.1016/j.fbio.2024.104235

[ref29] Taylor J. R. N. Dlamini B. C. Kruger J. (2013). 125th anniversary review: the science of the tropical cereals sorghum, maize and rice in relation to lager beer brewing. J. Inst. Brew. 119, 1–14. doi: 10.1002/jib.68

[ref30] Virkajärvi I. Vauhkonen T. Storgårds E. (2001). Control of microbial contamination in continuous primary fermentation by immobilized yeast. J. Am. Soc. Brew. Chem. 59, 63–68. doi: 10.1094/ASBCJ-59-0063

[ref9002] Wei Y. Zou W. Shen C. H. Yang J. G. (2020). Basic flavor types and component characteristics of Chinese traditional liquors: A review. J. Food Sci. 85, 4096–4107. doi: 10.1111/1750-3841.15536 33190291

[ref31] Wu F. Fan S. He G. Liang S. Xu Y. Tang K. (2024). Comparison of aroma compounds and sensory characteristics between two different types of rice-based baijiu. Foods 13:681. doi: 10.3390/foods13050681, 38472793 PMC10930833

[ref32] Wu H. Li W. Xin C. Zhang C. Wang Y. Ren S. . (2016). In vivo investigation to the macrolide-glycosylating enzyme pair DesVII/DesVIII in *Saccharopolyspora erythraea*. Appl. Microbiol. Biotechnol. 100, 2257–2266. doi: 10.1007/s00253-015-7036-9, 26552796

[ref33] Xu J. Wu H. Wang Z. Zheng F. Lu X. Li Z. . (2018). Microbial dynamics and metabolite changes in Chinese Rice wine fermentation from sorghum with different tannin content. Sci. Rep. 8:4639. doi: 10.1038/s41598-018-23013-1, 29545525 PMC5854674

[ref34] Xu Y. Zhao J. Liu X. Zhang C. Zhao Z. Li X. . (2022). Flavor mystery of Chinese traditional fermented baijiu: the great contribution of ester compounds. Food Chem. 369:130920. doi: 10.1016/j.foodchem.2021.130920, 34461518

[ref35] Yang Y. Hu W. Xia Y. Mu Z. Tao L. Song X. . (2020). Flavor formation in Chinese Rice wine (Huangjiu): impacts of the flavor-active microorganisms, raw materials, and fermentation technology. Front. Microbiol. 11:580247. doi: 10.3389/fmicb.2020.580247, 33281774 PMC7691429

[ref36] Yang F. Wang H. Chen L. Zhou N. Lu J. Pu X. . (2024). *Clostridium lapidicellarium* sp. nov. and *Clostridium moutaii* sp. nov., two species isolated from fermentation cellar-producing sauce-flavour Chinese baijiu. Int. J. Syst. Evol. Microbiol. 74:580. doi: 10.1099/ijsem.0.006580, 39560674 PMC11648564

[ref37] Yoshimoto H. Bogaki T. (2023). Mechanisms of production and control of acetate esters in yeasts. J. Biosci. Bioeng. 136, 261–269. doi: 10.1016/j.jbiosc.2023.06.009, 37607842

[ref38] Zhang J. Zhu X. Xu R. Gao Q. Wang D. Zhang Y. (2018). Isolation and identification of histamine-producing *Enterobacteriaceae* from Qu fermentation starter for Chinese rice wine brewing. Int. J. Food Microbiol. 281, 1–9. doi: 10.1016/j.ijfoodmicro.2018.05.014, 29800825

[ref39] Zhao Y. Gu M. Jiang P. Fang S. Yan N. Kong F. . (2024). Characterisation of aroma compounds, sensory characteristics, and bioactive components of a new type of huangjiu fermented with Chinese wild rice (*Zizania latifolia*). Food Chem. 452:139524. doi: 10.1016/j.foodchem.2024.139524, 38703742

[ref40] Zhou H. Yu Z. Yang G. Ji S. Zheng R. Liu Y. (2025). Understanding the application-related properties of ultrashort-duration varieties rice starch in different planting seasons. Int. J. Biol. Macromol. 314:144391. doi: 10.1016/j.ijbiomac.2025.144391, 40398783

[ref41] Zhu H. Yang H. Zhou X. Li H. Feng R. Yuan F. . (2023). Effect of DAP and glutamine supplementation on sulfur-containing volatiles and sensory properties of chardonnay wine fermented with *Saccharomyces cerevisiae* yeast. J. Food Sci. 88, 1392–1408. doi: 10.1111/1750-3841.16503, 36855306

[ref42] Zou J. Chen X. Wang C. Liu Y. Li M. Pan X. . (2023). Microbial communities and correlation between microbiota and volatile compounds in fermentation starters of Chinese sweet rice wine from different regions. Foods 12:2932. doi: 10.3390/foods12152932, 37569201 PMC10419015

